# Androgen and Cortisol Cosecreting Adrenal Adenoma and Tuberculous Lymphadenitis

**DOI:** 10.1210/jcemcr/luae171

**Published:** 2024-09-25

**Authors:** Gabriela Garza-García, José Diego Sánchez-Villa, Flavio Enrique Díaz-Trueba, Miguel Angel Lara-Salazar, Francisco Javier Gómez-Pérez, Alfredo Adolfo Reza-Albarrán

**Affiliations:** Department of Endocrinology, Instituto Nacional de Ciencias Médicas y Nutrición Salvador Zubirán, 14080 Mexico City, Mexico; Department of Endocrinology, Instituto Nacional de Ciencias Médicas y Nutrición Salvador Zubirán, 14080 Mexico City, Mexico; Department of Endocrine Surgery, Instituto Nacional de Ciencias Médicas y Nutrición Salvador Zubirán, 14080 Mexico City, Mexico; Department of Pathology, Instituto Nacional de Ciencias Médicas y Nutrición Salvador Zubirán, 14080 Mexico City, Mexico; Department of Endocrinology, Instituto Nacional de Ciencias Médicas y Nutrición Salvador Zubirán, 14080 Mexico City, Mexico; Department of Endocrinology, Instituto Nacional de Ciencias Médicas y Nutrición Salvador Zubirán, 14080 Mexico City, Mexico

**Keywords:** adrenal adenoma, 18F-FDG PET/CT, tuberculous lymphadenitis, adrenalectomy

## Abstract

The differential diagnosis between malignant and benign adrenal cortical tumors is challenging, and concurrent androgen and cortisol production should raise  suspicion of a malignant tumor. We present the case of a 36-year-old woman who exhibited pronounced hirsutism, clitoromegaly, and secondary amenorrhea. A contrast-enhanced computed tomography (CT) scan revealed a 35 × 27 mm right adrenal mass with unenhanced CT attenuation of 40 Hounsfield units (HUs). The mass exhibited absolute and relative washout rates of 50% and 28%, respectively, and was accompanied by a 25 × 20 mm adenopathy located in the hepatogastric space. Total testosterone was elevated by 247 ng/dL (8.56 nmol/L) (normal reference range, 10-75 ng/dL; 0.34-2.6 nmol/L). A 1-mg dexamethasone suppression test revealed an elevated serum morning cortisol concentration of 10.57 μg/dL (291.58 nmol/L) (reference range, <1.8 μg/dL; < 49.66 nmol/L). A fluorine-18 fluorodeoxyglucose positron emission tomography/computed tomography (18F-FDG PET/CT) scan revealed increased uptake in both the adrenal mass and the adenopathy. Subsequently, the patient underwent an open right adrenalectomy and lymphadenectomy. Histological examination revealed the presence of an adrenal adenoma with myelolipomatous metaplasia, as well as a positive polymerase chain reaction (PCR) for *Mycobacterium tuberculosis* in the adenopathy.

## Introduction

The number of endocrinology consultations for adrenal incidentalomas is increasing due to the widespread use of imaging studies. The initial evaluation is aimed at determining whether the adrenal mass is malignant and whether it is hormonally active. Although the majority of adrenal incidentalomas are benign lesions, there are biochemical and imaging data, such as hormonal cosecretion, unenhanced computed tomography (CT) attenuation of > 20 Hounsfield units (HUs), absolute < 60% and relative < 40% washout and increased fluorodeoxyglucose (FDG) uptake [1, 2], that are suspicious for a malignant lesion. We evaluated an adrenal incidentaloma that was found to secrete androgens and cortisol and had all of the previously mentioned suspicious imaging characteristics, along with an adenopathy in the hepatogastric space (between the liver and the lesser curvature of the stomach) that nonetheless was found to be an adrenal adenoma with concurrent tuberculous lymphadenitis due to excess cortisol.

## Case Presentation

A 36-year-old woman was referred for evaluation due to a right adrenal mass found on a CT scan performed during the workup for low back pain. She had no prior medical conditions and reported experiencing secondary amenorrhea since the age of 30. Additionally, since that age, she noted increased body hair on her forearms and face, which was managed with laser hair removal. The score reported prior to the aesthetic treatment was 27 points on the Ferriman-Gallwey scale, which is indicative of severe hirsutism.

## Diagnostic Assessment

**Figure 1.**
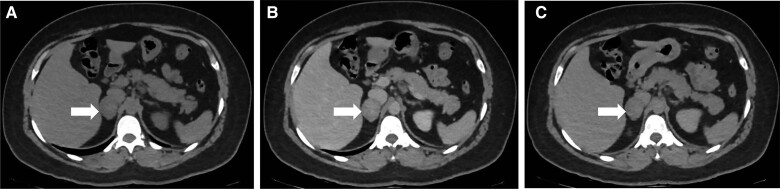
Axial images from an unenhanced and contrast-enhanced computed tomography scan showing a lipid-poor 35 × 27 mm right adrenal mass (arrows). A) Unenhanced attenuation of 40 Hounsfield units (HUs). B) Contrast-enhanced venous phase attenuation of 90 HUs. C) Delayed contrast-enhanced attenuation of 65 HUs.

**Figure 2.**
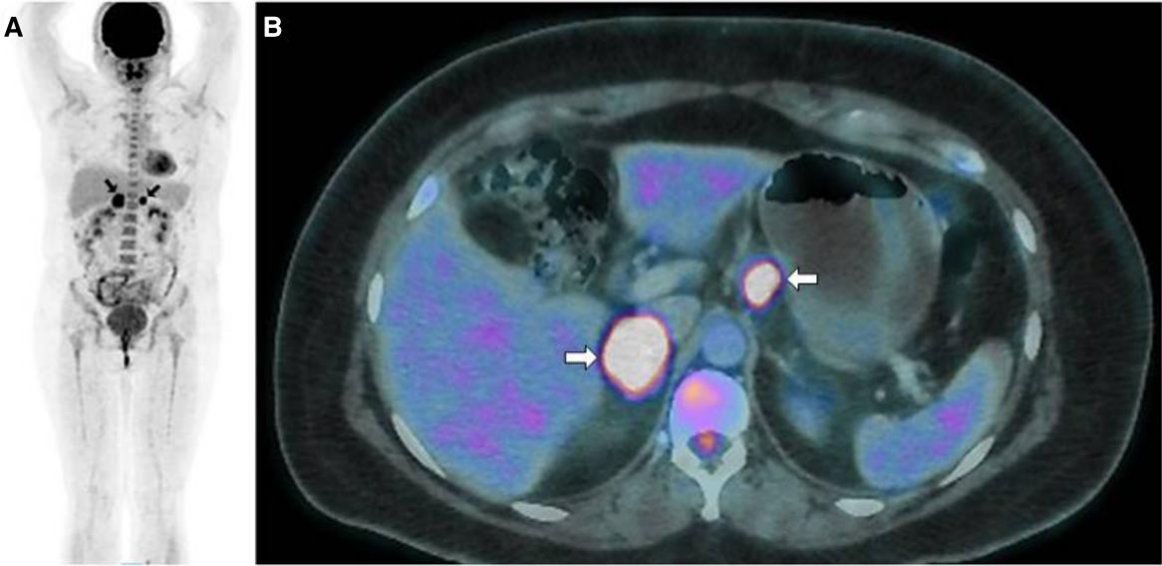
F-18 fluorodeoxyglucose positron emission tomography (18F-FDG PET/CT) image. A) Whole-body scan showing increased uptake in both the right adrenal mass and the solid nodular tissue in the hepatogastric space (dark arrows), but no lesions were observed in the liver or lungs. B) Axial slice from fused 18-FDG PET/CT showing that the right adrenal mass was markedly hypermetabolic, as was the nodular tissue in the hepatogastric space (white arrows), with standard uptake value (SUV) of 10.6 and 10.9, respectively.

During the physical examination, the patient presented with class I obesity, acne, a male pattern distribution of pubic hair and clitoromegaly. A contrast-enhanced CT scan was performed, which revealed a solid right adrenal mass measuring 35 × 27 mm with an attenuation of 40 HUs in the noncontrast phase, 90 HUs in the venous phase, and 65 HUs in the delayed phase. The scan showed an absolute washout of 50% and a relative washout of 28%. Additionally, there was solid nodular tissue in the hepatogastric space measuring 25 × 20 mm (Fig. 1). In the endocrine workup, biochemical hyperandrogenism and mild autonomous cortisol secretion (MACS) were found. Serum testosterone was elevated to 247 ng/dL (8.56 nmol/L) (normal reference range, 10-75 ng/dL; 0.34-2.6 nmol/L), as was androstenedione > 10 ng/mL (34.91 nmol/L) (normal reference range, 0.4-3.4 ng/mL; 1.39-11.87 nmol/L). The concentrations of dehydroepiandrosterone sulfate (DHEA-S) were within the normal range: 310 µg/dL (8.41 µmol/L) (normal reference range, 35-430 µg/dL; 0.94-11.67 µmol/L), as were those of dehydroepiandrosterone (DHEA): 8.3 ng/mL (28.80 nmol/L) (normal reference range, 1.65-13.5 ng/mL; 5.72-46.84 nmol/L) and 17 α-hydroxyprogesterone 3.1 ng/mL (9.38 nmol/L) (normal reference range, 0.95-5.0 ng/mL; 2.87-15.13 nmol/L). The beta subunit of human chorionic gonadotropin was undetectable. Due to the marked increase in serum testosterone and normal DHEA-S level, a transvaginal ultrasound was performed, which ruled out an ovarian tumor and showed polycystic ovarian morphology. A 1-mg overnight dexamethasone suppression test revealed a baseline serum cortisol concentration of 11.52 μg/dL (317.79 nmol/L) (normal reference range, 6.7-22.6 μg/dL; 184.82-623.44 nmol/L) and absence of suppression with serum cortisol 10.57 μg/dL (291.58 nmol/L) (reference range, < 1.8 μg/dL; < 49.66 nmol/L) after the test. The patient's adrenocorticotropic hormone (ACTH) levels were within the normal range: 30 pg/mL (6.61 pmol/L) (normal reference range, 10-100 pg/mL; 2.20-22.02 pmol/L). In addition to obesity, the patient's comorbidity associated with MACS was prediabetes with a glycated hemoglobin (A1C) level of 5.7% (39 mmol/mol). No vertebral fractures were detected in the CT scan, and bone densitometry was normal, with Z scores of 1.3 for the femoral neck, 1.7 for the total hip and 0.8 for the total lumbar spine. Fractionated metanephrines in urine and plasma were within normal ranges. Her serum potassium level was normal at 4.36 mmol/L (reference range, 3.5-5.1 mmol/L), and her blood pressure was normal. Due to the characteristics suggesting malignancy in the right adrenal mass, the adenopathy in the hepatogastric space was suspected to be due to metastasis. Therefore, fluorine-18 (18F)-FDG positron emission tomography/computed tomography (PET/CT) was performed and revealed a right adrenal mass measuring 28 × 34 mm with focal hypermetabolism with standard uptake value (SUV) of 10.6. The adenopathy in the hepatogastric space was also found to have focal increased metabolism (SUV of 10.9), and no lesions were observed in the liver or the lungs (Fig. 2).

## Treatment

Because of the cosecretion of cortisol and androgens and suspicious imaging characteristics, a right adrenalectomy with an open approach was performed, along with lymphadenectomy of the suspicious lymph node in the hepatogastric space. During the anesthesia induction, a stress dose of hydrocortisone was administered (100 mg intravenously as a bolus followed by 200 mg intravenously in continuous infusion over 24 hours). There were no complications during the surgery, and she was discharged with a regimen of hydrocortisone tapering.

## Outcome and Follow-Up

**Figure 3.**
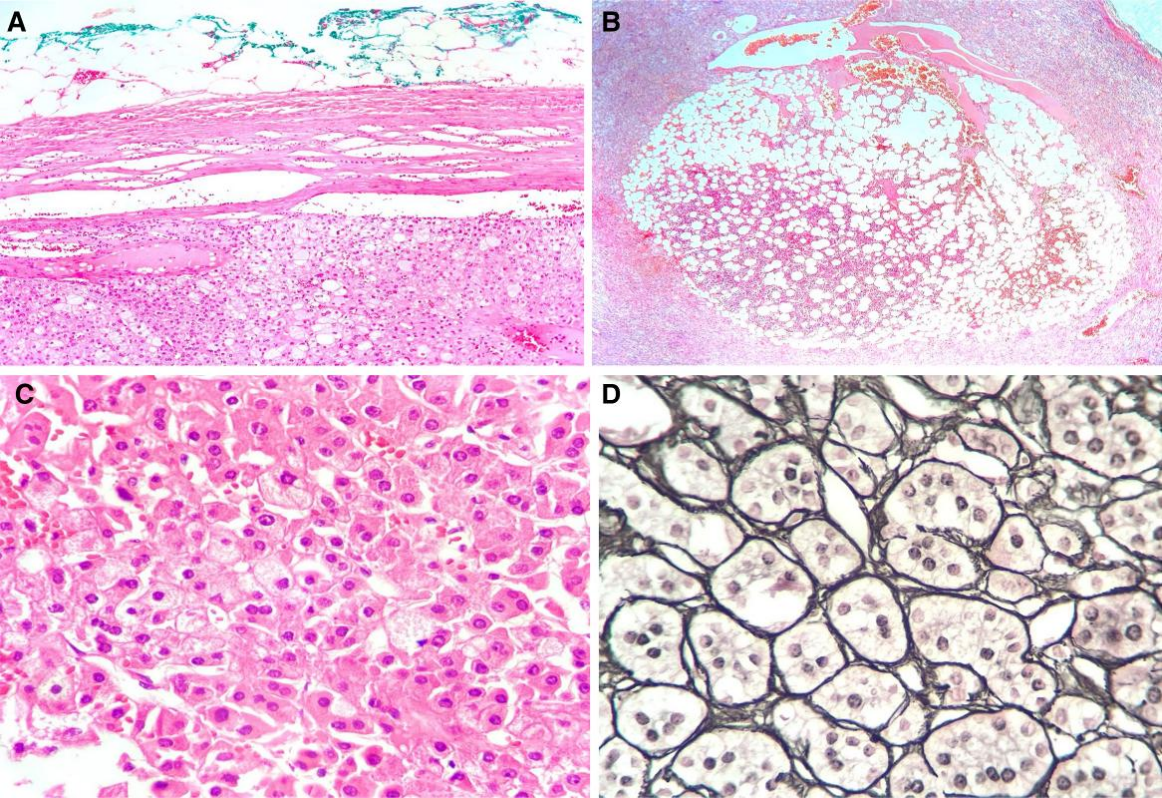
A) Hematoxylin and eosin (H&E) slide of the adrenal mass showing an encapsulated and well-delimitated lesion with a solid arrangement. B) H&E slide showing the adrenal adenoma and, in the center, a focus of myelolipomatous metaplasia. C) H&E staining at 40× magnification showing large adenoma cells arranged in solid sheets. The cells had a pink cytoplasm, and the round nuclei were centrally located with some nucleoli. D) H&E slide showing reticulin histochemical staining with an intact reticulin framework.

**Figure 4.**
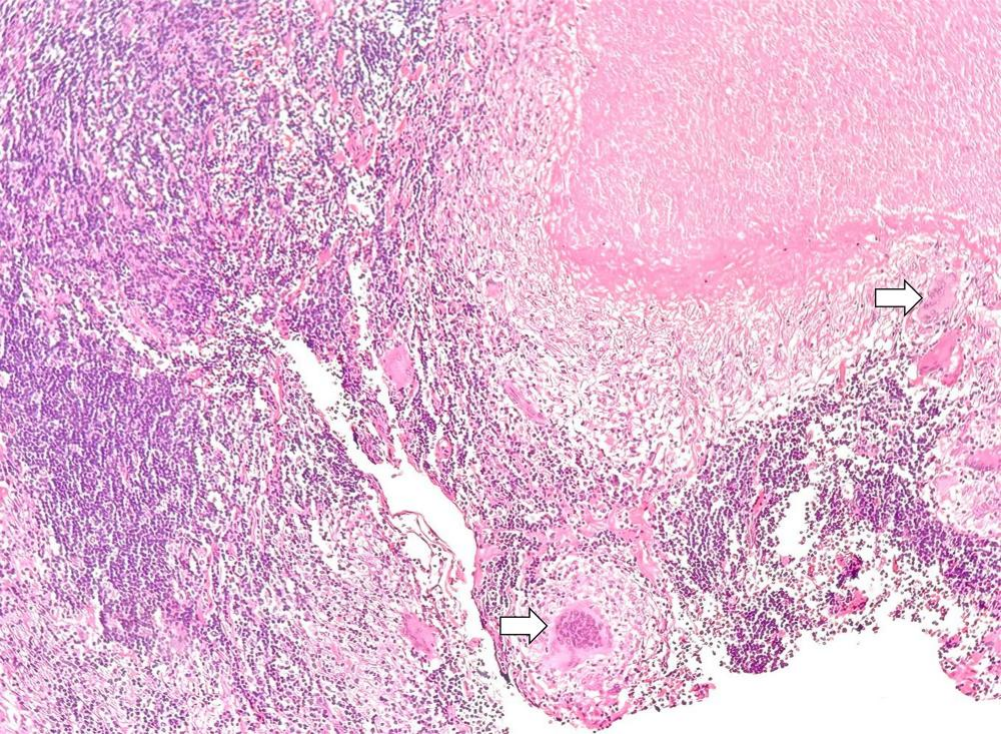
H&E staining showing the replacement of the lymph node architecture by areas of necrosis from granulomatous inflammation with multinucleated giant cells (white arrows).

The histopathological report of the right adrenalectomy revealed an adrenocortical adenoma with myelolipomatous metaplasia. The Weiss score was 2 points, the Ki67 labeling index was 2%, and the mass showed an intact reticulin framework (Table 1, Fig. 3). Histopathological examination of the lymph node revealed granulomatous lymphadenitis with caseous necrosis and Langhans-type multinucleated giant cells, which were suggestive of an infectious etiology, and staining to identify microorganisms (Ziehl-Neelsen) was negative (Fig. 4). However, the result of PCR testing of the adenopathy for *Mycobacterium tuberculosis* was positive. A chest x-ray obtained postadrenalectomy showed no signs of tuberculosis. During outpatient follow-up 4 weeks later, the patient reported no fever, chills, or cough while taking 15 mg of hydrocortisone daily in 2 divided doses. To assess the hypothalamic-pituitary-adrenal (HPA) axis, she was instructed to discontinue the daily hydrocortisone dosage 24 hours prior to blood collection for serum cortisol measurement. The resulting serum cortisol levels were indicative of ongoing suppression of the HPA axis: serum cortisol 3.61 μg/dL (99.5 nmol/L) (normal reference range, 6.7-22.6 μg/dL; 184.82-623.44 nmol/L) and ACTH 10 pg/mL (2.2 pmol/L) (normal reference range, 10-100 pg/mL; 2.20-22.02 pmol/L). Therefore, the replacement dose of hydrocortisone was continued, and instructions were given to manage malaise and vomiting. An infectious disease consultation was requested, and treatment with isoniazid 75 mg every 24 hours, rifampin 150 mg every 24 hours, pyrazinamide 400 mg every 24 hours, and ethambutol 300 mg every 24 hours was initiated. Four weeks after rifampin initiation, the patient exhibited a lower serum cortisol concentration than at the previous visit: a serum cortisol concentration of 1.86 μg/dL (51.31 nmol/L) and an ACTH concentration of 32 pg/mL (7.04 pmol/L) were associated with orthostatic symptoms. This was attributed to the induction of cytochrome P450 by rifampin, leading to increased cortisol metabolism. Consequently, the hydrocortisone dose was increased to 25 mg daily (15 mg in the morning and 10 mg in the afternoon), which led to an improvement in the patient's symptoms. Eighteen weeks after adrenalectomy, she showed signs of recovery in the HPA axis, with an increase in serum cortisol to 8.47 μg/dL (233.65 nmol/L) and ACTH to 42 pg/mL (9.24 pmol/L). As a result, a dose reduction of hydrocortisone was initiated, ultimately leading to its discontinuation 24 weeks after adrenalectomy. After the surgery, the patient's serum levels of testosterone and androstenedione returned to normal, with testosterone of 35 ng/dL (1.21 nmol/L) (normal reference range, 10-75 ng/dL; 0.34-2.6 nmol/L) and androstenedione of 2.11 ng/mL (7.36 nmol/L) (normal reference range, 0.4-3.4 ng/mL; 1.39-11.87 nmol/L), and she resumed her menstrual cycles. Twelve months after the surgery, the patient underwent follow-up imaging with a contrast-enhanced CT scan without evidence of recurrence. She has since remained asymptomatic and has completed the maintenance phase of tuberculosis treatment.

**Table 1.** Weiss score of the androgen and cortisol cosecreting adrenal adenoma

Weiss system	Patient's histopathological report
High nuclear grade	No, 0 points
> 5 mitoses per 50 high power field	0/50 high power field, 0 points
Atypical mitotic figures	No, 0 points
< 25% of tumor cells are clear cells	Yes, 2 points
Diffuse architecture (> 33% of tumor)	No, 0 points
Necrosis	No, 0 points
Venous invasion (smooth muscle in wall)	No, 0 points
Sinusoidal invasion (no smooth muscle in wall)	No, 0 points
Capsular invasion	No, 0 points

## Discussion

Cosecretion of androgens and cortisol is rare in adrenal adenomas [3]. We found 9 cases reported in the literature (Table 2), which suggests that the cosecretion of androgens and cortisol by an adrenal mass should not necessarily be considered a sign of malignancy. Interestingly, the basal ACTH level was not suppressed despite autonomous cortisol secretion. We have previously reported that the measurement of ACTH at our hospital is not reliable for distinguishing adrenal adenomas as the cause of hypercortisolism. In our retrospective series, only 20% of patients had ACTH values < 5 pg/mL (1.10 pmol/L) despite an adrenal source of hypercortisolism [11]. Distinguishing between malignant and benign adrenal cortical tumors is challenging. In this case, the long-term presentation of secondary amenorrhea and virilization, alongside the absence of metastasis, correlates with the pathology report indicating a benign adrenal tumor. Interestingly, the adrenal adenoma was combined with an adrenal myelolipoma, a rare but reported association in the literature [12]. This case also illustrates the diagnostic pitfalls of imaging studies. Among the imaging data suspicious for malignancy is attenuation > 20 HUs in the noncontrast phase; however, false positives, such as this case, can be explained by lipid-poor adenomas. Absolute and relative washout percentages (< 60% and < 40%, respectively) have been proposed as suspicious indicators for malignancy. However, their diagnostic efficacy in distinguishing benign from malignant lesions is only moderate, as suggested by a recent study [13]. In indeterminate adrenal masses, 18F-FDG PET/CT is suggested for distinguishing benign from malignant lesions. However, some benign lesions, such as cortisol-secreting adenomas, exhibit increased uptake [14]. The reason for the variable uptake among adenomas remains unclear, but it may be linked to their glucose metabolism and functional state [15]. This case illustrates the false positives of 18F-FDG, one of which was a functioning adrenal adenoma and the other of which was lymphadenopathy that turned out to be secondary to *Mycobacterium tuberculosis* infection. To the best of our knowledge, there is no association between tuberculosis and adrenal adenomas; nonetheless, unilateral adrenal incidentalomas as a presentation of adrenal tuberculosis have been reported [16, 17], suggesting that tuberculosis as a differential diagnosis should be considered when facing a unilateral adrenal incidentaloma. The indications for surgery for adrenal incidentalomas include a clinically significant hormonal excess or a risk of malignancy. In this patient, both a clinically significant hormonal excess (androgen secretion) and a risk of malignancy (> 20 HU, 18F-FDG PET/CT scan with increased uptake in the adrenal lesion and adenopathy) were detected, and surgical removal of the right adrenal gland was performed. In cases of suspected adrenal malignancy, unilateral adrenalectomy via an open transperitoneal approach is recommended as the standard treatment. However, in centers with sufficient experience, a laparoscopic approach may be performed for lesions < 6 cm without local invasion. Nonetheless, along with the endocrine surgery team, we decided to pursue an open approach because if the mass proved to be an adrenal adenocarcinoma, incomplete resection or tumor capsule rupture could compromise overall survival [18]. According to the hospital protocol, a stress dose of hydrocortisone was administered before anesthetic induction, and the dose was subsequently tapered. The recovery of the HPA axis was faster than that reported in some cases of Cushing syndrome due to adrenal adenoma, likely due to the milder cortisol secretion, shorter duration of symptoms, and the patient's young age [19]. In this case, a significant event was the increase in cortisol metabolism induced by hepatic microsomal enzyme induction by rifampicin. There have been case reports in the literature of adrenal crisis precipitated by this mechanism, highlighting the need for close monitoring during the recovery of the HPA axis [20]. Finally, this case illustrates that rapid-onset hirsutism associated with testosterone concentrations > 150 ng/dL (5.2 nmol/L) should raise suspicion of an ovarian or adrenal tumor as the underlying etiology [21]. In addition, in this case, the excess testosterone originated from the adrenal glands and possibly induced polycystic changes in the patient's ovaries, a situation previously observed in transgender men [22].

**Table 2.** Published cases of androgen and cortisol cosecreting adrenal adenoma

First author, year	Study type	Patient characteristics		Laboratory evaluation						Abdominal CT scan	Surgical management
		Assigned sex at birth, age, and presentation	Total testosterone	Androstenedione	DHEA-S	DHEA	17(OH)P	ACTH	Serum cortisol after dexamethasone suppression test		
Murakami Y [4], 1995	Case report	AFAB 28 years old Hirsutism Secondary amenorrhea	↑ 507.61 ng/dL (17.6 nmol/L) (8.65 -57.68 ng/dL; 0.3-2 nmol/L)	NR	↑ 423.72 μg/dL (11.5 μmol/L) (reference range, 51.58-298.45 μg/dL; 1.4-8.1 μmol/L)	NR	NR	↓<9.08 pg/mL (<2 pmol/L) (reference range NR)	↑ 0.5 mg dexamethasone every 6 hours (2 mg/day) for 7 days: 11.96 μg/dL (330 nmol/L)	Round tumor with a diameter of 3 cm in the left adrenal region	Left adrenalectomy
Delgrange E [5], 1996	Case report	AFAB 68 years old Hirsutism, alopecia, hypertension	↑ 111.04 ng/dL (3.85 nmol/L) (reference range, <69.22 ng/dL; <2.4 nmol/L)	↑ 3.58 ng/mL (12.5 nmol/L) (reference range, 1.43-2.57 ng/mL; 5-9 nmol/L),	↑ 420.04 μg/dL (11.4 μmol/L) (reference range, 92.11-221.07 μg/dL; 2.5-6 μmol/L)	↑ 4.63 ng/mL (16.1 nmol/L) (reference range, 2.01-4.61 ng/mL; 7-16 nmol/L)	↔ 0.49 ng/mL (1.5 nmol/L) (reference range, 0.33-1.65 ng/mL; 1-5 nmol/L)	↓ 11 pg/mL (2.42 pmol/L) (reference ,0-100 pg/mL; 4.40-22.02 pmol/L)	↑ 2 mg dexamethasone every 12 hours for 7 days: 8:00 Am serum cortisol 6.23 μg/dL (172 nmol/L)	Bilateral heterogeneous adrenal masses with a diameter of 6 cm on the right side and of 3 cm on the left side No HUs provided	Bilateral adrenalectomy
Danilowicz K [6], 2002	Case report	AFAB 33 years old Hirsutism, weight gain	↑ 150 ng/dL (5.20 nmol/L) (reference range, 30-80 ng/dL; 1.04-2.77 nmol/L)	NR	↑ 495 μg/dL (13.43 μmol/L) (reference range, 65-380 μg/dL; 1.76-10.31 μmol/L)	NR	NR	↔/↓<20 pg/mL (<4.40 pmol/L) (reference range, 9-52 pg/mL; 1.98-11.45 pmol/L)	↑ 1 mg overnight dexamethasone suppression test serum cortisol 14 μg/dL (386 nmol/L) 8 mg dexamethasone suppression test serum cortisol before: 14.1 μg/dL (388.96 nmol/L) and after 8 mg dexamethasone 12 μg/dL (331.03 nmol/L)	Right adrenal mass	Right adrenalectomy
Danilowicz K [6], 2002	Case report	AFAB Postmenopausal women (age not specified) Hirsutism, deepening of her voice	↑ 670 ng/dL (23.23 nmol/L) (reference range, 30-80 ng/dL; 1.04-2.77 nmol/L)	NR	↑ 934.4 μg/dL (25.35 μmol/L) (reference range, 65-380 μg/dL; 1.76-10.31 μmol/L)	NR	NR	NR	↑ 1 mg overnight dexamethasone suppression test serum cortisol 7.4 μg/dL (204.13 nmol/L)	Large (6 cm) left adrenal mass	Left adrenalectomy
Ambrosi B [7], 2008	Letter to the editor	AFAB 33 years old Alopecia	↑ 181 ng/dL (6.27 nmol/L) (reference range, <100 ng/dL; <3.46 nmol/L)	↑ 6.3 ng/mL (21.9 nmol/L) (reference range, 0.3-3.3 ng/mL; 1.04-11.52 nmol/L)	↔ 120 μg/dL (3.25 μmol/L) (reference range, 35-430 μg/dL; 0.94-11.67 μmol/L)	NR	↑ 16 ng/mL (48.41 nmol/L) (reference range, 0.2-1.8 ng/mL; 0.60-5.44 nmol/L)	↓<5 pg/mL (1.1 pmol/L) (reference range NR)	↑ 1-mg overnight dexamethasone suppression test serum cortisol 9.9 μg/dL (273.10 nmol/L)	Ovoidal tumor of 4.7 cm, not homogeneous and with some small calcifications, in the left adrenal region No HUs provided	Left laparoscopic adrenalectomy
Tanaka S [8], 2011	Case report	AFAB 53 years old Acne, hirsutism, clitoromegaly, full plethoric face, central obesity, purple-red abdominal striae, proximal muscle weakness	↑ 189.6 ng/dL (6.57 nmol/L) (reference range, 6-82 ng/dL; 0.20-2.84 nmol/L)	NR	↑ 602 μg/dL (16.33 μmol/L) (reference range, 11-116 μg/dL; 0.29-3.14 μmol/L)	NR	NR	↓<5 pg/mL (1.10 pmol/L) (reference range, 10-60 pg/mL; 2.20-13.21 pmol/L)	↑ 1 mg overnight dexamethasone suppression test serum cortisol 22.4 μg/dL (617.93 nmol/L)	Left adrenal mass measuring 4.0 × 3.0 × 2.5 cm in largest dimensions No HUs provided	Left laparoscopic adrenalectomy
Tanaka S [8], 2011	Case report	AFAB 26 years old Secondary amenorrhea, axillary and abdominal purple-red striae, and emotional lability	NR	NR	↑ 356 μg/dL (9.66 μmol/L) (reference range, 44-332 μg/dL; 1.19 -9.01 μmol/L)	NR	NR	↓<1 pg/mL (0.22 pmol/L) (reference range, 10-60 pg/mL; 2.20-13.21 pmol/L)	↑ 8 mg dexamethasone suppression test serum cortisol 19 μg/dL (524.13 nmol/L)	Right adrenal mass measuring 3.1 × 2.0 × 2.0 cm, homogenously enhanced by contrast administration No HUs provided	Right laparoscopic adrenalectomy
Alqahtani A [9], 2022.	Abstract	AFAB 35 years old Symptoms of androgen excess along with Cushing features not otherwise specified	↑ 62.87 ng/dL (2.18 nmol/L) (reference range, 10.96-56.81 ng/dL; 0.38-1.97 nmol/L)	NR	NR	↑ 22 umol/L (reference range, 2-11.1 umol/L)	NR	↔ 5.35 pg/mL (1.18 pmol/L) (reference range, 4.67-48.58 pg/mL; 1.03-10.70 pmol/L)	↑ 1 mg overnight dexamethasone suppression test serum cortisol 17.8 μg/dL (493 nmol/L)	Heterogeneous left adrenal mass (4.4 × 7 × 4.6 cm) with enhancing solid and cystic/necrotic components and small round calcifications No HUs provided	Left laparoscopic adrenalectomy
Kitamura T [10], 2022	Case report	AFAB 41 years old Hypertension, male pattern hair loss	↑ 119 ng/dL (4.12 nmol/L) (reference range, 15-44 ng/dL; 0.52-1.52 nmol/L)	↑ 3.31 ng/mL (11.55 nmol/L) (reference range, 0.76-1.40 ng/mL; 2.65-4.88 nmol/L)	↑ 2250 μg/dL (61.06 μmol/L) (reference range, 41-218 μg/dL; 1.11-5.91 μmol/L)	↑ 6.87 ng/mL (23.83 nmol/L) (reference range, 1.55-4.03 ng/mL; 5.37-13.98 nmol/L)	↔ 0.46 ng/mL (1.39 nmol/L) (reference range, 0.04-2.74 ng/mL; 0.12-8.29 nmol/L)	↓<1.5 pg/mL (<0.33 pmol/L) (reference range NR)	↑ 1-mg overnight dexamethasone suppression test serum cortisol 12 μg/dL (331 nmol/L)	6-cm left adrenal tumor with well-defined borders, unenhanced mean HUs of the adrenal tumor was 19.1 and contrast enhancement showed heterogeneous appearance of the tumor	Left laparoscopic adrenalectomy

Abbreviations: 17(OH)P, 17α hydroxyprogesterone; ACTH, adrenocorticotropic hormone; AFAB, assigned female at birth; CT, computed tomography; DHEA, dehydroepiandrosterone; DHEA-S, dehydroepiandrosterone sulfate; Hus, Hounsfield unit; NR, not reported.

## Learning Points

- The differential diagnosis between malignant and benign adrenal cortical tumors represents a significant challenge. Thus, a comprehensive evaluation incorporating clinical, imaging, and pathological characteristics is imperative for precise diagnosis.
- The cosecretion of androgens and cortisol from an adrenal incidentaloma should raise suspicion of adrenocortical carcinoma and lead to adrenalectomy. However, in some cases, such cosecretion may also be due to an adrenal adenoma.
- False positives in 18F-FDG PET/CT can be caused by a functioning adrenal adenoma or by infections, especially in the context of cortisol excess.
- The hepatic microsomal enzyme-inducing effect of rifampicin causes an increase in cortisol metabolism; therefore, the hydrocortisone dose should be increased for patients receiving replacement therapy.

## Data Availability

Some or all datasets generated during and/or analyzed during the current study are not publicly available but are available from the corresponding author on reasonable request.

## Abbreviations

18F-FDG PET/CT: fluorine-18 fluorodeoxyglucose positron emission tomography/computed tomography
ACTH: adrenocorticotropic hormone
CT: computed tomography
DHEA-S: dehydroepiandrosterone sulfate
FDG: fluorodeoxyglucose
HPA: hypothalamic-pituitary-adrenal
HU: Hounsfield unit
MACS: mild autonomous cortisol secretion
SUV: standard uptake value
